# Real-world dosing of regorafenib and outcomes among patients with metastatic colorectal cancer: a retrospective analysis using US claims data

**DOI:** 10.1186/s12885-024-12421-4

**Published:** 2024-08-02

**Authors:** Tanios Bekaii-Saab, Nasreen Khan, Helene Ostojic, XiaoLong Jiao, Guifang Chen, Wenlong Lin, Amanda Bruno

**Affiliations:** 1 Mayo Clinic, 5777 E. Mayo Blvd, Phoenix, AZ 85054 USA; 2 Bayer HealthCare Pharmaceuticals, 100 Bayer Blvd, Whippany, NJ 07981 USA; 3 Bayer Consumer Care, Peter Merian-Strasse 84, Basel, 4052, Switzerland; 4 Present Address: Pfizer, 66 Hudson Blvd, New York, NY USA; 5 Present Address: Syneos Health, 100 Brandywine Blvd, Newtown, PA 18940 USA

**Keywords:** Real world, Dosing, Regorafenib, Outcomes, Metastatic colorectal cancer, Retrospective, Claims data, United States

## Abstract

**Background:**

The randomized, dose-optimization, open-label ReDOS study in US patients with metastatic colorectal cancer (CRC) showed that, compared with a standard dosing approach, initiating regorafenib at 80 mg/day and escalating to 160 mg/day depending on tolerability increased the proportion of patients reaching their third treatment cycle and reduced the incidence of adverse events without compromising efficacy. Subsequently, the ReDOS dose-escalation strategy was included as an alternative regorafenib dosing option in the National Comprehensive Cancer Network (NCCN) Clinical Practice Guidelines. A retrospective analysis was conducted using a US claims database to assess whether inclusion of this dose-escalation strategy in NCCN Guidelines has influenced the use of flexible dosing in routine US clinical practice, and to describe clinical outcomes pre- and post-inclusion in NCCN Guidelines.

**Methods:**

Patients with CRC in the Optum’s de-identified Clinformatics® Data Mart database initiating regorafenib for the first time between January 2016 and June 2020 were stratified based on whether they initiated regorafenib pre- or post-inclusion of ReDOS in NCCN Guidelines, and in two groups: flexible dosing (< 160 mg/day; < 84 tablets in the first treatment cycle) and standard dosing (160 mg/day; ≥ 84 tablets in the first treatment cycle). The primary endpoints were the proportion of patients who initiated their third treatment cycle and the mean number of treatment cycles per group.

**Results:**

703 patients initiated regorafenib during the study period, of whom 310 (44%) initiated before and 393 (56%) initiated after inclusion of ReDOS in NCCN Guidelines. After inclusion in the guidelines, the proportion of patients who received flexible dosing increased from 21% (*n* = 66/310) to 45% (*n* = 178/393), the proportion who received standard dosing decreased from 79% (*n* = 244/310) to 55% (*n* = 215/393), the proportion who initiated their third treatment cycle increased from 36% (*n* = 113/310) to 46% (*n* = 179/393), and the mean (standard deviation) number of treatment cycles increased from 2.6 (2.9) to 3.2 (3.1).

**Conclusions:**

Following inclusion of ReDOS in NCCN Guidelines, real-world data suggest that US clinicians have markedly increased use of flexible dosing in clinical practice, potentially maximizing clinical benefits and safety outcomes for patients with metastatic CRC receiving regorafenib.

**Supplementary Information:**

The online version contains supplementary material available at 10.1186/s12885-024-12421-4.

## Background

Colorectal cancer (CRC) is the fourth most common cancer diagnosed in the USA, with 152,810 estimated new cases (7.6% of all new cancer cases) and 53,010 estimated deaths (8.7% of all cancer-related deaths) in 2024 [1]. The 5-year relative survival rate of metastatic CRC (mCRC) in the USA is 16% [1]. The mortality associated with metastatic disease highlights the unmet need for effective treatments for these patients [2].

Current standard-of-care therapies for mCRC include fluoropyrimidine-based chemotherapy and anti-vascular endothelial growth factor (VEGF) or anti-epidermal growth factor receptor (EGFR) treatments for patients with *RAS* wild-type disease [3–5]. These options are the standard backbone early-line systemic treatments for mCRC, with multiple options for refractory disease [2]. Regorafenib is an oral multikinase inhibitor approved as a third- or later-line option for previously treated patients with advanced mCRC, advanced gastrointestinal stromal tumors (GISTs), or unresectable hepatocellular carcinoma (HCC) at a dosage of 160 mg/day for 3 weeks on/1 week off (standard dosing) [6]. Regorafenib was approved for the treatment of patients with mCRC who have been previously treated with fluoropyrimidine-, oxaliplatin-, and irinotecan-based chemotherapy, an anti-VEGF therapy, and, if *RAS* wild-type, an anti-EGFR therapy [6]. This approval was based on the results of the pivotal randomized, double-blind, placebo-controlled phase III CORRECT trial (NCT01103323), which showed an overall survival benefit for regorafenib versus placebo (median 6.4 vs. 5.0 months) when combined with best supportive care [7]. The safety of regorafenib was subsequently evaluated in a larger population in the international phase IIIb single-arm study CONSIGN (NCT01538680), which reported consistent frequency and severity of adverse events and similar median progression-free survival [8].

Regorafenib has an acceptable and predictable safety profile, but adverse events (AEs) occurring early in treatment, such as hand–foot skin reaction and fatigue, may have limited its optimum use in the initial years following approval [9]. Consequently, several prospective studies have evaluated the effect of different dosing schedules on the safety and efficacy of regorafenib, and these studies suggest that starting regorafenib at a lower-than-label-recommended (flexible) dose with the aim of escalating to 160 mg/day is feasible and can lead to better treatment tolerability and longer duration of therapy [9–12]. In an international, prospective observational safety study of regorafenib in real-world practice (CORRELATE; *N* = 1037), 12% of patients with mCRC initiated treatment at 80 mg/day, 30% at 120 mg/day, and 57% at the approved dosage of 160 mg/day. Although almost half of patients received a starting dose less than the standard dose, rates of some treatment-related AEs were lower than in CORRECT, including hand–foot skin reaction (26% vs. 47%), whereas median overall survival and progression-free survival were similar [11]. A dose-optimization, randomized, phase II study in the USA comparing regorafenib standard dose with a first cycle dose-escalation strategy (ReDOS; NCT02368886) further confirmed that patients with mCRC could initiate regorafenib at 80 mg/day and then escalate to 160 mg/day depending on tolerability [9]. In this study, the dose-escalation approach increased the proportion of patients reaching their third treatment cycle and reduced the incidence of AEs without compromising efficacy. In addition, the primary endpoint was met, with 23/54 patients (43%) in the dose-escalation group completing two treatment cycles and initiating a third treatment cycle versus 16/62 patients (26%) in the standard-dose group [9]. Subsequently, in March 2018, the ReDOS dose-escalation strategy was included as an alternative regorafenib dosing option in the National Comprehensive Cancer Network (NCCN) Clinical Practice Guidelines for colon and rectal cancers [3, 4].

To the best of the authors’ knowledge, there are no real-world data on how the ReDOS strategy has influenced clinical practice and outcomes, and this study aimed to address this gap in knowledge regarding current patterns of use of regorafenib in US clinical practice. To this end, a retrospective analysis using a US claims database was used to assess whether inclusion of the ReDOS dose-escalation strategy in NCCN Guidelines has influenced the use of flexible dosing in the first treatment cycle in routine US clinical practice as measured by trends in regorafenib treatment patterns (low starting dose vs. standard dosing), and to describe clinical outcomes. Further specific objectives were to describe the proportion of patients who initiated their third treatment cycle and the mean number of treatment cycles pre- and post-inclusion of the ReDOS strategy in the NCCN Guidelines.

## Methods

### Study design and patients

**Fig. 1:**
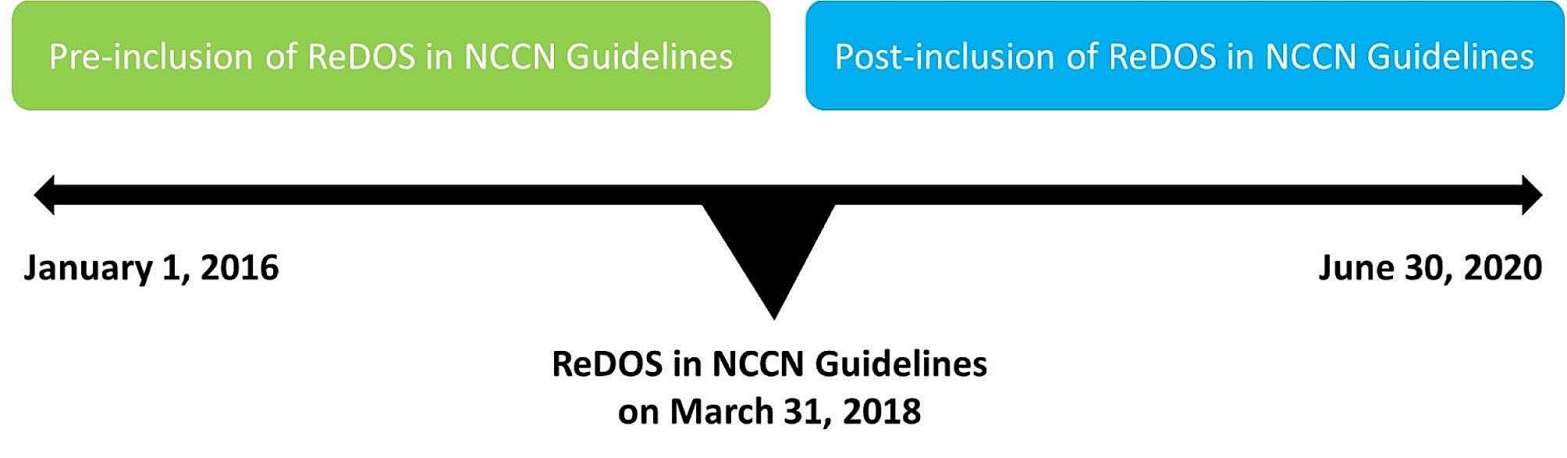
Study design

This was a retrospective, observational cohort study of patients with mCRC in the Optum’s de-identified Clinformatics® Data Mart database who initiated regorafenib for the first time (index date) between January 1, 2016, and June 30, 2020 (Fig. 1). The Clinformatics® database contains health insurance claims data across inpatient and outpatient services, as well as prescription drug and enrollment information from a national private insurance provider. This database covers a proportion of the commercially insured and Medicare Advantage population in all 50 US states and Washington DC and is considered representative of the broader US commercially insured population in terms of age and gender [13]. The database includes filled prescription-level data, including tablet quantities, days of supply, and date the prescription was filled [13].

Patients aged ≥ 18 years with a diagnosis of CRC were included. Regorafenib is also approved for the treatment of patients with GISTs or HCC, who are likely different from those with CRC. Therefore, patients who had a diagnosis of GIST or HCC ≤ 6 months before the index date (baseline period) were excluded to avoid potential selection bias. Patients had ≥ 6 months of insurance coverage before the index date and for ≥ 3 months thereafter (the post-index period was until end of insurance eligibility or data cut-off or death [patients who died < 3 months after the index date were included in the analysis]). Patients were stratified based on whether they initiated regorafenib pre- or post-inclusion of ReDOS in the NCCN Guidelines (as of March 31, 2018), and in two groups based on the number of regorafenib 40 mg tablets filled in their first 28-day treatment cycle: flexible dosing, defined as < 84 tablets, indicating that the patient initiated regorafenib at lower dosages (80 or 120 mg/day or a different combination) than the approved standard dosage; or standard dosing, defined as ≥ 84 tablets (160 mg/day).

### Endpoints and assessments

The primary endpoints were the proportion of patients who initiated their third treatment cycle (a composite endpoint that encapsulates safety and activity parameters) and the mean number of treatment cycles per group (a cycle was defined as patients receiving regorafenib once daily for the first 21 days of each 28-day period [i.e., 3 weeks on/1 week off]). In addition, exploratory analyses were conducted to evaluate clinical outcomes according to timing of regorafenib initiation, defined as pre- or post-inclusion of the ReDOS strategy in the NCCN Guidelines, stratified by flexible dosing versus standard dosing and characteristics of patients who reached their third treatment cycle versus those who did not.

### Statistical analysis

The study was descriptive in nature and no hypotheses were tested; all patients who met the study inclusion criteria were included in the analyses. Descriptive analysis was conducted using Statistical Analysis System (Version 9.4 [TS1M5]) software. In addition, several sensitivity analyses were conducted: analysis restricted to patients who had no other primary cancer (not specified in the exclusion criteria) at baseline (except skin cancer); cut-off period extended by 3 months (to June 30, 2018) to account for potential delays between guideline publication and practice adoption; and dose definition modified to account for patients who were not filling their prescription in accordance with the 28-day cycle.

### Sensitivity analyses

Sensitivity analyses were carried out that included (1) patients without other primary cancers at baseline (except skin cancer); (2) a ReDOS cut-off date of June 2018 (3 months after publication of revised NCCN Guidelines that included the ReDOS dose-escalation strategy) to account for potential delays in practice changes; (3) dose modification for a subset of patients who were not filling their prescription in accordance with the 28-day cycle.

## Results

### Patient characteristics

A total of 703 patients received regorafenib during the study period and were included in the final analysis (overall study population): 310 (44.1%) before and 393 (55.9%) after inclusion of ReDOS in NCCN Guidelines. Patient demographics and clinical characteristics, including prior treatments, were similar in patients who initiated regorafenib pre- and post-inclusion of ReDOS in NCCN Guidelines. There were some differences in geographic region (more patients from the Midwest [28.4% vs. 18.3%] and fewer patients from the South [42.9% vs. 52.7%]) and mean follow-up time (8.6 vs. 6.9 months) in the pre-ReDOS inclusion group compared with the post-ReDOS inclusion group (Table 1).

**Table 1:** Patient demographics and clinical characteristics at regorafenib treatment initiation (pre- or post-inclusion of the ReDOS strategy in NCCN Guidelines)

**Characteristic**	Study population (*N* = 703)	
	Pre-inclusion of ReDOS in NCCN Guidelines (*n* = 310)	Post-inclusion of ReDOS in NCCN Guidelines (*n* = 393)
Age, years		
Mean (SD)	63.1 (12.3)	65.2 (11.3)
Median (range)	63 (34.0–89.0)	67 (32.0–89.0)
Mean, *n* (%)	175 (56.5)	220 (56.0)
Race, *n* (%)		
African American/Black	39 (12.6)	40 (10.2)
Asian	14 (4.5)	19 (4.8)
Hispanic	39 (12.6)	42 (10.7)
Unknown	17 (5.5)	70 (17.8)
White	201 (64.8)	222 (56.5)
US region, *n* (%)		
Midwest	88 (28.4)	72 (18.3)
Northeast	26 (8.4)	36 (9.2)
South	133 (42.9)	207 (52.7)
West	63 (20.3)	78 (19.8)
Payer category, *n* (%)*		
Commercial	153 (49.4)	150 (38.2)
Medicare Advantage	157 (50.6)	243 (61.8)
Charlson Comorbidity Index		
Mean (SD)	2.1 (2.2)	2.4 (2.2)
Median (range)	2.0 (0–13.0)	2.0 (0–15.0)
Prior hospitalizations, *n* (%)	100 (32.3)	122 (31.0)
Mean number of visits (SD)	1.5 (0.8)	1.4 (0.8)
Hand–foot skin reaction at baseline, *n* (%)	55 (17.7)	73 (18.6)
Hypertension at baseline, *n* (%)	179 (57.7)	249 (63.4)
Anti-EGFR treatment at baseline, *n* (%)	82 (26.5)	87 (22.1)
Anti-VEGF treatment at baseline, *n* (%)	143 (46.1)	181 (46.1)
Chemotherapy at baseline, *n* (%)	252 (81.3)	301 (76.6)
Trifluridine/tipiracil treatment at baseline, *n* (%)	62 (20.0)	74 (18.8)
Immunotherapy at baseline, *n* (%)	< 5 (–)^†^	9 (2.3)
Follow-up time, months		
Mean (SD)	8.6 (9.0)	6.9 (5.5)
Median (range)	5.5 (0–47.7)	5.5 (0–29.7)

*The Clinformatics® database covers a proportion of the commercially insured and Medicare Advantage population in all 50 US states and Washington DC. Medicare is US medical insurance for eligible patients, including those aged ≥ 65 years or who have certain disabilities or conditions. Medicare beneficiaries may be covered under a traditional Medicare plan (government managed) or the Medicare Advantage plan (managed by private companies approved by Medicare); ^†^Values < 5, or values that could be used to derive such values, have been suppressed to protect patient confidentiality

EGFR, epidermal growth factor receptor; NCCN, National Comprehensive Cancer Network; SD, standard deviation; VEGF, vascular endothelial growth factor

### Regorafenib dosing patterns

The use of flexible dosing increased post- versus pre-inclusion of ReDOS in NCCN Guidelines (45.3% vs. 21.3%) (Table 2). In addition, the proportion of patients reaching their third treatment cycle and the mean number of treatment cycles were higher post- than pre-inclusion of ReDOS in NCCN Guidelines (Table 2).

**Table 2:** Clinical outcomes according to time of regorafenib treatment initiation (pre- or post-inclusion of the ReDOS strategy in NCCN Guidelines)

Clinical characteristic	Study population (*N* = 703)	
	Pre-inclusion of ReDOS in NCCN Guidelines (*n* = 310)	Post-inclusion of ReDOS in NCCN Guidelines (*n* = 393)
Dose classification at index date, *n* (% [95% CI])*		
Flexible dose (< 84 tablets/28 days^†^)	66 (21.3 [16.9, 26.3])	178 (45.3 [40.3, 50.4])
Standard dose (≥ 84 tablets/28 days)	244 (78.7 [73.7, 83.1])	215 (54.7 [49.6, 59.7])
Patients reaching their third treatment cycle, *n* (% [95% CI])	113 (36.5 [31.1, 42.1])	179 (45.5 [40.5, 50.6])
Number of treatment cycles		
Mean (SD)	2.6 (2.9)	3.2 (3.1)
Median (range)	2.0 (0.5–27.0)	2.0 (0.5–26.0)

*Each tablet contained regorafenib 40 mg; ^†^Administration of < 84 tablets/28 days indicates that the patient initiated regorafenib at a lower than standard dose (80 or 120 mg/day, or a different combination)

CI, confidence interval; NCCN, National Comprehensive Cancer Network; SD, standard deviation

Overall, 292/703 patients received a third treatment cycle, with a higher proportion in the post-ReDOS inclusion group (179/393; 45.5%) versus the pre-ReDOS inclusion group (113/310; 36.5%). In an exploratory analysis, most patient demographics and clinical characteristics were similar between patients reaching their third treatment cycle and those who did not, although patients who did not reach their third treatment cycle had more prior hospitalizations, and a greater proportion had received anti-VEGF treatment and chemotherapy at baseline (Supplemental Table S1).

Table 3 presents clinical outcomes in the overall study population and the exploratory/subgroup analysis by dose group (flexible vs. standard dosing). The proportion of patients reaching their third treatment cycle increased similarly from the pre- to post-ReDOS inclusion period in both the flexible dosing (34.8% vs. 44.9%) and standard dosing (36.9% vs. 46.0%) groups, whereas there was a greater increase in the mean number of treatment cycles in the flexible dosing group (2.3 vs. 3.3) compared with the standard dosing group (2.7 vs. 3.1) (Table 3).

**Table 3:** Clinical outcomes according to time of regorafenib treatment initiation (pre- or post-inclusion of the ReDOS strategy in NCCN Guidelines), stratified by dose group

Clinical characteristic	Pre-inclusion of ReDOS in NCCN Guidelines (*n* = 310)		Post-inclusion of ReDOS in NCCN Guidelines (*n* = 393)	
	Standard dosing (*n* = 244)	Flexible dosing (*n* = 66)	Standard dosing (*n* = 215)	Flexible dosing (*n* = 178)
Number of treatment cycles				
Mean (SD)	2.7 (3.2)	2.3 (1.7)	3.1 (3.0)	3.3 (3.2)
Median (range)	2.0 (1.0–27.0)	2.0 (0.5–11.0)	2.0 (1.0–26.0)	2.0 (0.5–20.0)
Patients reaching the third treatment cycle, *n* (% [95% CI])	90 (36.9 [30.8, 43.3])	23 (34.8 [23.5, 47.6])	99 (46.0 [39.2, 53.0])	80 (44.9 [37.5, 52.6])

CI, confidence interval; NCCN, National Comprehensive Cancer Network; SD, standard deviation

### Sensitivity analyses

After excluding patients with other primary cancers (*n* = 193), 510 patients were available for a sensitivity analysis that included patients without other primary cancers at baseline (except skin cancer). Patient demographics and clinical characteristics were similar to those of the overall study population. Overall, median Charlson Comorbidity Index scores (predictor of 10-year survival in patients with multiple comorbidities) were lower in this sensitivity analysis cohort (1.00) versus the overall study population (2.00), and more patients in the overall study population (31.6%) versus the sensitivity analysis cohort (25.7%) were hospitalized. Similar dosing pattern results to the overall study population were observed (Supplemental Table S2). The proportion of patients reaching their third treatment cycle, the mean number of treatment cycles, and the proportion of patients using flexible dosing were similar between the overall study population and those without other primary cancers. These results held true when the ReDOS cut-off date was moved from March 2018 to June 2018 to account for potential delays in practice changes (Supplemental Table S3). In the third sensitivity analysis, the dose was modified for a subset of patients (*N* = 670) who were not filling their prescription in accordance with the 28-day cycle; again, outcomes were similar to those seen in the overall population (Supplemental Table S4).

## Discussion

### Uptake of flexible (ReDOS) dosing strategies in US clinical practice

The ReDOS study showed that a first cycle dose-escalation strategy with regorafenib for patients with mCRC reduced the incidence of AEs without compromising efficacy [9]. The aim of this real-world investigation was to determine how the ReDOS strategy has influenced US clinical practice and clinical outcomes in patients with mCRC. In this study, the flexible dosing group comprised patients who initiated regorafenib at lower than standard dosages of 80 mg/day or 120 mg/day. We observed a 24% increase in the use of flexible dosing (< 84 tablets) in the first treatment cycle post-inclusion of ReDOS in the NCCN Guidelines and a 24% decrease in the use of standard dose (≥ 84 tablets).

### Outcomes following flexible dosing in US clinical practice

The proportion of patients who reached their third treatment cycle was 9% higher post- versus pre-inclusion of ReDOS in NCCN Guidelines. The mean (standard deviation) number of treatment cycles was 2.6 (2.9) pre-inclusion of ReDOS in NCCN Guidelines and 3.2 (3.1) post-inclusion. The observed increases in flexible dosing were accompanied by increases in the mean number of treatment cycles by approximately 20%, with nearly half of patients reaching their third treatment cycle in both the flexible dosing and the standard dosing groups after NCCN guidelines included the ReDOS dose-escalation strategy – this finding may suggest a customized dosing strategy tailored to individual patients’ disease status and needs. The results of this study were also robust in the different sensitivity analyses. These analyses supported an increased use of flexible dosing post-ReDOS inclusion, with patients more frequently reaching their third treatment cycle and having a higher mean number of treatment cycles. The most common reasons for patients failing to initiate a third cycle of regorafenib treatment are disease progression and adverse events [9]. Therefore, third treatment cycle initiation is a key endpoint in clinical trials of regorafenib evaluating dose escalation strategies because it considers the toxicity that most frequently occurs early in treatment (usually in the first cycle and improving thereafter [9]) and the drug’s effectiveness as measured by patients’ continued treatment beyond their second scan (Cycle 2) tumor assessment. The proportion of patients initiating a third cycle of therapy thus represents a composite endpoint that encapsulates safety and activity parameters; therefore, these findings represent a clinically relevant and valuable contribution to therapeutic decision-making with the potential to improve survival outcomes and quality of life.

### Comparison with prior studies

Previous studies have supported the concept of flexible dosing of regorafenib in patients with mCRC [9–11]. A numerically lower incidence of grade 3/4 AEs was seen with flexible dosing (defined as regorafenib 120 mg/day 3 weeks on/1 week off or 160 mg/day 1 week on/1 week off in the first treatment cycle followed by standard dosing thereafter) in the phase II REARRANGE study, which also showed efficacy results consistent with those of phase III studies [7, 10, 14]. In addition, efficacy was also reported with lower regorafenib starting doses in the CORRELATE prospective study [11] and in a Japanese study in patients who initiated regorafenib at 120 mg/day [15]. Furthermore, a systematic review and network meta-analysis evaluating different dosing strategies for regorafenib in refractory mCRC revealed that a flexible regorafenib dose-escalation strategy (defined as regorafenib 80 mg/day with weekly dose escalation if no significant drug-related toxicities, up to 160 mg/day) was superior to best supportive care, and showed a trend in survival benefit compared with regorafenib standard dosing [16].

### Adoption of flexible dosing and implications for clinical practice

According to the findings of the present study, clinicians in the USA have adopted this strategy in clinical practice. Clinical outcomes of the proportion of patients reaching the third treatment cycle and the number of treatment cycles were increased with both standard and flexible dosing post-inclusion of ReDOS in NCCN Guidelines, suggesting customized dosing strategies have been implemented. This increased use of flexible dosing in the first cycle, potentially combined with expected better patient selection and AE management over time, appears to have led to an improvement in treatment duration with regorafenib for many patients. A similar strategy has influenced clinical decision-making in South America, where a focus on flexible dosing in the early stages of treatment has helped ensure that patients reach their optimum dose based on tolerability [17]. The findings from the current study may further help inform routine clinical practice in US patients. A personalized and flexible approach to dose escalation is likely to be particularly important for patients who are older and/or have a poor health status, who would otherwise not have been able to benefit from regorafenib treatment [17, 18].

### Key strengths of the study

The present study has several key strengths, particularly the use of a US claims database, which includes data from a large single payer system – United Healthcare – with representation of the US population and detailed drug prescription information. Moreover, United Healthcare is the largest provider of Medicare Advantage but also provides non-Medicare commercial insurance. This population is therefore expected to comprise both Medicare Advantage patients (including those aged ≥ 65 years or disabled) and patients aged < 65 years covered by United Healthcare (i.e., provided by employers). This has enabled the retrospective description of demographics, clinical characteristics, and treatment patterns among a broad representation of US patients initiating regorafenib prescriptions before and after the inclusion of the ReDOS dose-escalation strategy in NCCN Guidelines. Moreover, no previous studies have used a large representative database to assess clinical practice changes before and after NCCN Guideline updates in the USA. Insurance eligibility was required to ensure that patients had not been previously treated with regorafenib and to provide baseline characteristics. Similarly, post-index (continuous) eligibility criteria were required to ensure a minimum follow-up time. In addition, in this closed-claims analysis, filling of prescriptions was captured regardless of US region. As the survival time of patients with CRC can be short, those who died within 3 months of eligibility were included so as not to bias the results towards patients with a better health status.

### Study limitations

This study has some limitations that warrant cautious interpretation of the results, and as with any retrospective claims-based association study, misclassification bias due to coding or transformation errors cannot be excluded. Moreover, inherent selection bias is a feature of the study because the reasons individual patients are initiated on lower than standard doses of regorafenib are unknown but assumed to be based on tolerability. An untested assumption is that initiating on a lower dose is not a function of health status; therefore, an overestimation of the impact of flexible dosing attributable to clinicians starting patients on lower doses due to poor health cannot be ruled out.

An additional limitation of the study is that since the Clinformatics® data are primarily representative of the US commercially insured and Medicare Advantage population, the study may not fully represent the population of patients with mCRC treated with regorafenib who are uninsured or underinsured, and/or in countries outside the USA. It should also be noted that there may have been potential confounding factors that were not captured in claims records. Another limitation is that although prescription fills are accurately recorded, patient adherence to therapy is not. To mitigate potential selection and misclassification bias and missed prescription filling, sensitivity analyses examined the restriction of patients without other primary cancers, alternative cut-off date demarcations for pre- and post-inclusion of ReDOS in NCCN Guidelines, and a modified dose definition to account for variable time in prescription fills, and similar results were observed.

## Conclusions

In conclusion, these real-world data suggest that clinicians in the USA have increased their use of flexible dosing and have adopted the ReDOS dose-escalation strategy in clinical practice, potentially improving the tolerability and duration of regorafenib treatment in patients with mCRC. Additional real-world observational studies, including those based on inferential statistics, are needed to confirm these findings, and future investigations are required to quantify how later-line therapy sequencing, coupled with flexible dosing, can maximize clinical benefit and safety outcomes for patients with mCRC. This may be important for clinical decision-making on behalf of patients in later lines of therapy, particularly those who are older and/or have poor health status, to potentially enable them to benefit from all available treatments, including regorafenib.

### Electronic supplementary material

Below is the link to the electronic supplementary material.

Supplementary Material 1

## Data Availability

The data that support the findings of this study are available from Optum, which were used under license for the current study, and so are not publicly available. Interested researchers should contact Optum (https://www.optum.com*).*

## Abbreviations

AE: Adverse event
CRC: Colorectal cancer
mCRC: Metastatic colorectal cancer
NCCN: National Comprehensive Cancer Network
