# Supplementary material for: Real-world dosing of regorafenib and outcomes among patients with metastatic colorectal cancer: a retrospective analysis using US claims data
Source: BMC Cancer. 2024 Aug 2;24:939. doi: 10.1186/s12885-024-12421-4 (PMC11295488; doi:10.1186/s12885-024-12421-4)
Supplement: Supplementary file 1 — Supplementary Material 1 [file 12885_2024_12421_MOESM1_ESM.docx]

**Additional files**

**Additional file 1 Patient demographics and clinical characteristics of patients who did or did not reach their third treatment cycle**

| **Characteristic** | **Reached third treatment cycle  (*n*=292)** | **Did not reach third treatment cycle (*n*=411)** | **All patients (*N*=703)** |
| --- | --- | --- | --- |
| Age, years  Mean (SD)  Median (range) | 63.9 (11.3)  65 (32.0–89.0) | 64.5 (12.1)  67 (34.0–88.0) | 64.3 (11.8)  66 (32.0–89.0) |
| Male, *n* (%) | 164 (56.2) | 232 (56.4) | 396 (56.3) |
| Race, *n* (%)  African American/Black  Asian  Hispanic  Unknown  White | 38 (13.0)  9 (3.1)  32 (11.0)  42 (14.4)  171 (58.6) | 41 (10.0)  24 (5.8)  49 (11.9)  45 (10.9)  252 (61.3) | 79 (11.2)  33 (4.7)  81 (11.5)  87 (12.4)  423 (60.2) |
| US region, *n* (%)  Midwest  Northeast  South  West | 63 (21.6)  29 (9.9)  145 (49.7)  55 (18.8) | 97 (23.6)  33 (8.0)  195 (47.4)  86 (20.9) | 160 (22.8)  62 (8.8)  340 (48.4)  141 (20.1) |
| Payer category, *n* (%)*  Commercial  Medicare Advantage | 131 (44.9)  161 (55.1) | 172 (41.8)  239 (58.2) | 303 (43.1)  400 (56.9) |
| Charlson Comorbidity Index  Mean (SD)  Median (range) | 2.2 (2.1)  2.0 (0–10.0) | 2.3 (2.3)  2.0 (0–15.0) | 2.3 (2.2)  2.0 (0–15.0) |
| Prior hospitalizations, *n* (%)  Mean number of visit  (SD) | 82 (28.1)  1.4 (0.7) | 140 (34.1)  1.5 (0.8) | 222 (31.6)  1.4 (0.8) |
| Hand–foot skin reaction at baseline, *n* (%) | 53 (18.2) | 75 (18.2) | 128 (18.2) |
| Hypertension at baseline, *n* (%) | 176 (60.3) | 252 (61.3) | 428 (60.9) |
| Anti-EGFR treatment at baseline, *n* (%) | 73 (25.0) | 96 (23.4) | 169 (24.0) |
| Anti-VEGF treatment at baseline, *n* (%) | 113 (38.7) | 211 (51.3) | 324 (46.1) |
| Chemotherapy at baseline, *n* (%) | 219 (75.0) | 334 (81.3) | 553 (78.7) |
| Trifluridine/tipiracil treatment at baseline, *n* (%) | 57 (19.5) | 79 (19.2) | 136 (19.3) |
| Immunotherapy at baseline, *n* (%) | 5 (1.7) | 5 (1.2) | 10 (1.4) |
| Follow-up time, months  Mean (SD)  Median (range) | 10.5 (8.1)  8.0 (2–47.7) | 5.6 (5.9)  3.8 (0–41.3) | 7.7 (7.3)  5.5 (0–47.7) |

**Additional file 2 Clinical outcomes at time of regorafenib treatment initiation (pre- or post-inclusion of the ReDOS strategy in NCCN Guidelines) for patients without other primary cancers at baseline (except skin cancer)**

| **Clinical characteristic** | **Study population (*N*=510)** | |
| --- | --- | --- |
|  | **Pre-inclusion of ReDOS in NCCN Guidelines  (*n*=221)** | **Post-inclusion of ReDOS in NCCN Guidelines  (*n*=289)** |
| Dose classification at index date,  *n* (% [95% CI])*  Flexible dose (<84 tablets/28 days)  Standard dose (≥84 tablets/28 days) | 49 (22.2 [16.9, 28.2])  172 (77.8 [71.8, 83.1]) | 129 (44.6 [38.8, 50.6])  160 (55.4 [49.4, 61.2]) |
| Number of treatment cycles  Mean (SD)  Median (range) | 2.7 (3.0)  2.0 (0.5–27.0) | 3.2 (3.3)  2.0 (0.5–26.0) |
| Patients reaching their third treatment cycle, *n* (% [95% CI]) | 84 (38.0 [31.6, 44.8]) | 130 (45.0 [39.2, 50.9]) |

**Additional file 3 Clinical outcomes according to extended cut-off date (June 30, 2018)**

| **Clinical characteristic** | **Study population (*N*=703)** | |
| --- | --- | --- |
|  | **Pre-inclusion of ReDOS in NCCN Guidelines  (*n*=352)** | **Post-inclusion of ReDOS in NCCN Guidelines  (*n*=351)** |
| Dose classification at index date,  *n* (% [95% CI])*  Flexible dose (<84 tablets/28 days)  Standard dose (≥84 tablets/28 days) | 80 (22.7 [18.5, 27.5])  272 (77.3 [72.5, 81.5]) | 164 (46.7 [41.4, 52.1])  187 (53.3 [47.9, 58.6]) |
| Number of treatment cycles  Mean (SD)  Median (range) | 2.7 (2.9)  2.0 (0.5–27.0) | 3.3 (3.2)  2.0 (0.5–26.0) |
| Patients reaching the third treatment cycle, *n* (% [95% CI]) | 130 (36.9 [31.9, 42.2]) | 162 (42.6 [40.8, 51.5]) |

**Additional file 4 Clinical outcomes at time of regorafenib treatment initiation (pre- or post-inclusion of the ReDOS strategy in NCCN Guidelines) according to modified dose definition**

| **Clinical characteristic** | **Study population (*N*=670)** | |
| --- | --- | --- |
|  | **Pre-inclusion of ReDOS in NCCN Guidelines  (*n*=294)** | **Post-inclusion of ReDOS in NCCN Guidelines  (*n*=376)** |
| Dose classification at index date,  *n* (% [95% CI])*  Flexible dose (<84 tablets/28 days)  Standard dose (≥84 tablets/28 days) | 81 (27.6 [22.5, 33.0])  213 (72.4 [67.0, 77.5]) | 187 (49.7 [44.6, 54.9])  189 (50.3 [45.1, 55.4]) |
| Number of treatment cycles  Mean (SD)  Median (range) | 2.7 (3.2)  2.0 (0.5–29.8) | 3.3 (3.2)  2.0 (0.5–26.0) |
| Patients reaching the third treatment cycle, *n* (% [95% CI]) | 112 (38.1 [32.5, 43.9]) | 180 (47.9 [42.7, 53.1]) |
